# Effects of epimuscular myofascial force transmission on sarcomere length of passive muscles in the rat hindlimb

**DOI:** 10.14814/phy2.12608

**Published:** 2015-11-04

**Authors:** Chris Tijs, Jaap H van Dieën, Huub Maas

**Affiliations:** Department of Human Movement Sciences, Faculty of Behaviour and Movement Sciences, Vrije Universiteit AmsterdamMOVE Research Institute Amsterdam, The Netherlands

**Keywords:** Ankle, skeletal muscle mechanics, soleus, tibialis anterior

## Abstract

Results from imaging studies and finite element models suggest epimuscular myofascial effects on sarcomere lengths in series within muscle fibers. However, experimental evidence is lacking. We evaluated epimuscular myofascial effects on (1) muscle belly, fiber, and mean sarcomere length and (2) sarcomere length distribution within passive fibers of the rat tibialis anterior (TA) and soleus (SO) muscles. Hindlimbs (*n* = 24) were positioned in predefined knee (55°, 90°, 125°, 160°) and ankle (either 90° or 125°) angles, and fixed in a formaldehyde solution. Varying knee joint angle causes changes in muscle–tendon unit length of SO and TA’s synergists, but not of SO and TA. Whole fibers were taken from SO and TA and photographed along their length. Mean sarcomere length was assessed for the entire fiber and for the proximal, intermediate, and distal thirds (fiber segments) separately. Mean sarcomere length of the fiber was not affected by knee angle, neither for SO (mean: 2.44 ± 0.03 *μ*m and 2.19 ± 0.05 *μ*m for ankle angles of 90° and 125°, respectively) nor for TA (mean: 2.33 ± 0.05 *μ*m and 2.51 ± 0.07 *μ*m for ankle angle set to 90° and 125°, respectively). Only for TA, a significant interaction between knee angle and fiber segment was found, indicating changes in the distribution of lengths of in-series sarcomeres. Thus, while epimuscular myofascial force transmission did not cause mean sarcomere length changes within passive SO and TA, it did alter the length distribution of sarcomeres within passive TA.

## Introduction

Striated muscle contains muscle fibers that exist of many sarcomeres in series. During human and animal movement, muscles and hence muscle fibers change length. As already proposed by Hill (1953), it is unlikely that length changes of muscle fibers are uniformly distributed among sarcomeres. Because the force that a sarcomere can produce is dependent on its length (Huxley 1957), nonuniformities of sarcomere length have direct consequences for muscle mechanical function. It has been found that individual differences in the range in which active force can be exerted by the rat semimembranosus muscle can largely be explained by distribution of sarcomere lengths between muscle fibers (Willems and Huijing 1994). Any nonuniformities may also be detected by sensory receptors such as muscle spindles and, hence, could affect sensory encoding of local muscle conditions (Cameron et al. 1981).

Strain nonuniformities between muscle fibers were found in the toad semimembranosus (Ahn et al. 2003), in the turkey lateral gastrocnemius, and in the frog palmaris longus (Azizi and Deslauriers 2014). In the latter study, the strain nonuniformities between fibers of the turkey lateral gastrocnemius were explained by variation in pennation angle over the muscle. Nonuniformities between fibers of the frog palmaris longus were explained by the different location of the fibers (superficial vs. central). This suggests that these nonuniformities all could be explained exclusively by muscle architectural properties. In addition to nonuniformities between muscle fibers, nonuniform length changes of in-series sarcomeres have been reported within single frog muscle fibers (Julian and Morgan 1979; Burton et al. 1989), as a result of variations in active force generating capacity or passive stiffness of the in-series sarcomeres (Rassier 2012).

Many studies have shown that muscle fiber force can be transmitted not only via the muscles’ origin and insertion (i.e., myotendinous pathway), but also via linkages located at the muscles’ epimysial surface (i.e., epimuscular myofascial force transmission) (Huijing 2009; Maas and Sandercock 2010). Finite element (FE) models of skeletal muscles (Yucesoy 2010) predicted effects of epimuscular myofascial force transmission on distributions of sarcomere length between and within muscle fibers (Yucesoy et al. 2003, 2006). A characteristic feature of epimuscular myofascial force transmission is that, if a neighboring muscle is lengthened distally, the force of a length-restrained muscle increases proximally and decreases distally (Huijing 2009). For such a condition, an FE model predicted strain increases in the proximal part, representing an increase in sarcomere length, and strain decreases in the distal part, representing a decrease in sarcomere length (Yucesoy et al. 2003). However, these model predictions have not been validated.

Experimentally, a recent study showed with magnetic resonance imaging that passive knee extension (proximal lengthening of the gastrocnemius muscle relative to the soleus muscle) in humans caused local strains in passive soleus (SO) muscle (Huijing et al. 2011). It should be noted that these strains were not in the muscle fiber direction. Changes in muscle fiber length have been assessed with ultrasound imaging, but the results are contradicting. Knee extension was reported to cause shortening of passive SO muscle fibers (Tian et al. 2012), while others found that fascicle length of passive and active human SO was not affected by knee angle (Kawakami et al. 1998). Moreover, these studies assessed effects of epimuscular myofascial connections on length changes at the level of muscle fibers, while the effects on sarcomere lengths have not been investigated.

The overall aim of the present study was to investigate effects of epimuscular myofascial interactions between adjacent synergistic muscles on the length parameters of muscles within the anterior and posterior crural compartments of the rat. For that purpose, we assessed effects of passive muscle–tendon unit (MTU) length changes of muscles on (1) muscle belly, fiber, and mean sarcomere length and (2) length distribution of in-series arranged sarcomeres within passive muscle fibers of their monoarticular length-restrained synergists.

## Material and Methods

### Ethical approval

The hindlimbs used in the present study were obtained after experiments for a different study that was approved by the Committee on Ethics of Animal Experimentation at the VU University Amsterdam (Permit Number: FBW 13-03). All procedures were in agreement with the guidelines and regulations concerning animal welfare and experimentation set forth by Dutch law. According to standard procedures in our laboratory (Maas et al. 2001), intraperitoneally injected urethane was used to deeply anesthetize the animals, such that reflexes were fully suppressed. Without regaining conscious, these animals were euthanized with an overdose of pentobarbital sodium.

### Hindlimb fixation

Left and right hindlimbs (*n* = 24) of male Wistar rats (body mass 321.8 ± 34.2 g, mean ± SD) were shaved and separated from the body. The limbs were secured on a wooden plate at predefined knee angles (55°, 90°, 125°, 160°) for ankle angles set to either 90° (*n* = 12) or 125° (*n* = 12), and stored in a 3.7% formaldehyde solution for several days. By imposing different knee angles at a constant ankle angle (*n* = 3 for each limb configuration), monoarticular SO and tibialis anterior (TA) muscles were kept at a constant MTU length, while the length of their bi- and polyarticular synergists (i.e., gastrocnemius and plantaris for SO, extensor digitorum longus (EDL) for TA) was changed proximally.

### X-ray images of the hindlimb

Lateral X-ray images (X-ray tube: Toshiba E7865X, SEDECAL, Algete, Spain) of the fixed limbs were collected (60 kVp, 13 mAs, 100 msec). From these images, tibia length and actual joint angles were determined using ImageJ 1.47 (Rasband 1997). Joint angles were assessed in a comparable way as described earlier (Felder et al. 2005): ankle angle was defined as the included angle between the long axis of the calcaneus (anterior to the ankle joint) and the line from the mid-point of the distal tibia through the mid-point of the tibial plateau. Knee angle was defined by the included angle between the long axis of the femur and the above described line through the tibia plateau.

### Dissection and treatment of muscle fibers

SO (muscle mass: 122.2 ± 12.1 mg, mean ± SD) and TA (muscle mass: 608.3 ± 74.7 mg) muscles were dissected from the fixed hindlimbs, and muscle belly lengths were measured using calipers and normalized to tibia length (%L_M_T_). The muscles were rinsed in demineralized water for at least 2 days to remove the formaldehyde solution and, subsequently, treated with a 26% nitric acid (HNO_3_) solution for 4 h to partly digest intramuscular connective tissues. The muscles were then washed with demineralized water and stored in a 50% glycerol (C_3_H_8_O_3_) solution for several days until fiber dissection (Huijing and Maas 2015).

**Figure 1:**
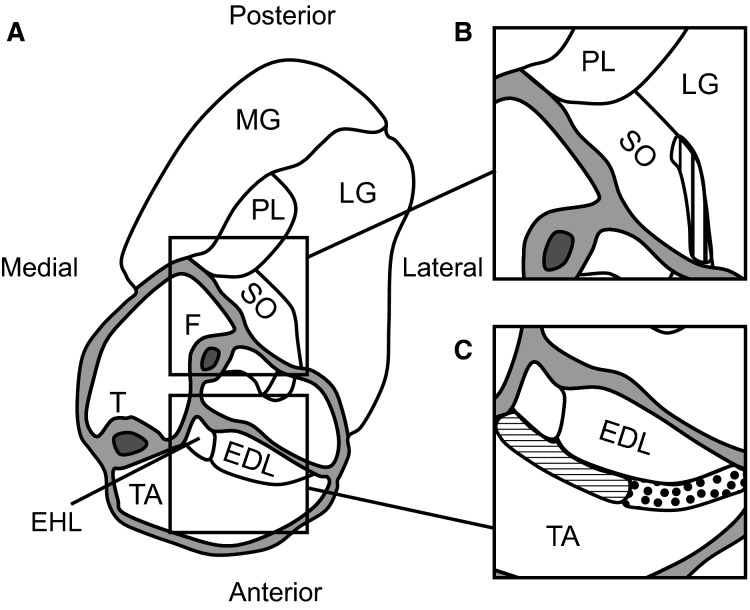
Schematic of a cross section of the right anterior and posterior crural compartments at approximately the distal third of the tibia. (A) Muscles in the posterior crural compartment (soleus, SO; plantaris, PL; medial and lateral gastrocnemius, MG and LG) and in the anterior crural compartment (tibialis anterior, TA; extensor digitorum longus, EDL; extensor hallucis longus, EHL) as well as the tibia (T) and fibula (F) are identified. (B) Detail of the lateral superficial SO–LG interface (vertical stripes) from which multiple whole SO fibers were dissected. (C) Detail of the superficial medial (horizontal stripes) and lateral (dotted) TA–EDL interface from which multiple whole TA fibers were dissected. Because the length of fibers that are located in the medial TA region were not equal to those in the lateral TA region, these fibers were analyzed separately. Cross section modified from Figure1D in Rijkelijkhuizen et al. (2007).

For SO, at least three whole muscle fibers were dissected from several areas close to the lateral interface between SO and lateral gastrocnemius (Fig.1). For TA, fibers originating within the proximal region of the medial interface between TA and EDL and inserting on the medial side of distal internal aponeurosis of TA appeared shorter than fibers originating close to the lateral TA–EDL interface and inserting of the lateral side of distal internal TA aponeurosis. Therefore, these regions were analyzed separately (Fig.1). Within each region at least three whole muscle fibers distributed over the region were dissected. Muscle fibers where then placed on a microscopic slide and photographed entirely as well as along their length from proximal to distal using a digital camera (MikroCam 5 MP, Bresser, Rhede, Germany) mounted on a microscope (Ortholux II, Leitz, Wetzlar, Germany). This resulted in one photograph for each fiber to assess fiber length, and in a series of photographs (∼30, magnification 3240×) for each fiber to assess sarcomere lengths.

### Assessment of fiber and sarcomere length

**Figure 2:**
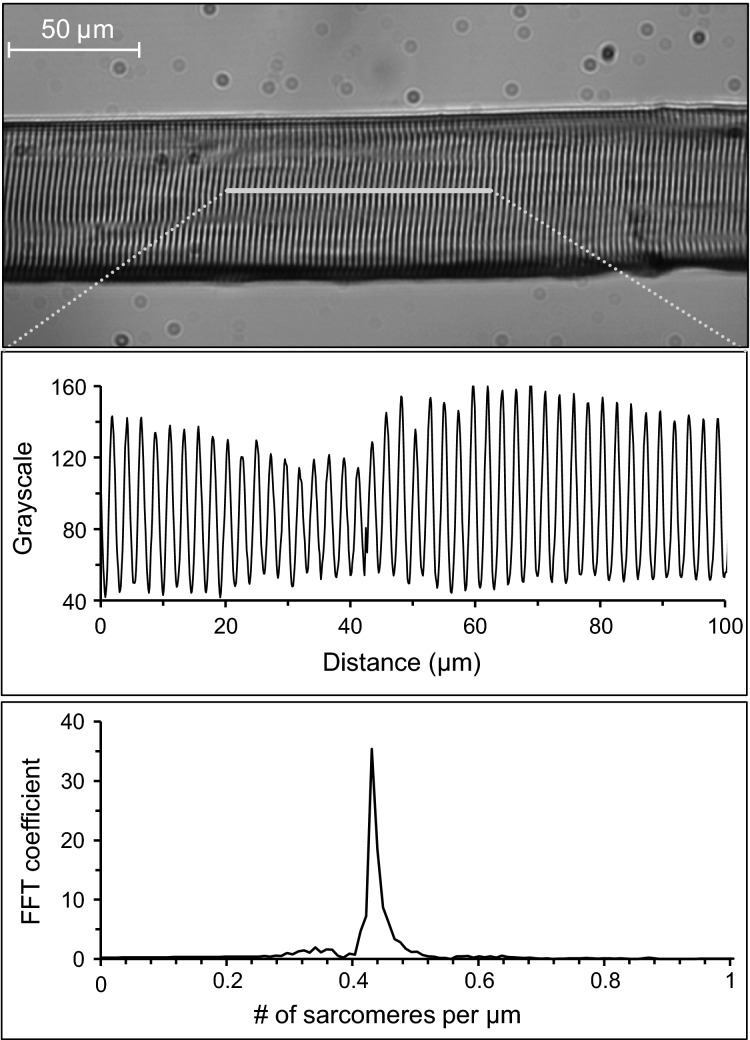
Procedures to assess sarcomere length. Photographs of muscle fiber sections were taken, region of interests were selected manually (upper panel), and the distribution of grayscale values were determined (middle panel). A discrete Fourier transform was used to calculate the number of sarcomeres per *μ*m (peak value in the bottom panel). Mean sarcomere length of the region of interest was calculated as 1/number of sarcomeres per *μ*m.

Fiber length was determined using ImageJ 1.47 (Rasband –2015^1997^) and normalized to tibia length (%L_F_T_). To assess sarcomere length, a region of interest was selected for each photograph and the corresponding distribution of gray scale values (0 = black, 255 = white) along the length of the fiber was determined using the “plot profile” function in ImageJ (Fig.2). This resulted in a sinusoidal waveform in which maxima and minima represent the I-bands and A-bands of the in-series sarcomeres, respectively. The gray scale signal was filtered (Butterworth, 5th order) to exclude sarcomere lengths above 3 *μ*m (cut-off frequency: 0.33 sarcomeres per *μ*m) and below 1.5 *μ*m (cut-off frequency: 0.67 sarcomeres per *μ*m). A discrete Fourier transform (MATLAB 2014a, Mathworks, Natick, MA) was performed to determine mean number of sarcomeres per *μ*m, from which the mean sarcomere length of the selected region of interest was calculated (1/number of sarcomeres per *μ*m).

Fiber mean sarcomere length was calculated by averaging the sarcomere lengths obtained from all photographs per fiber. To assess length distributions of sarcomeres, mean sarcomere lengths were calculated for the proximal, intermediate, and distal segments separately. Each segment included one third of the photographs of each fiber. Fiber mean sarcomere length and segment mean sarcomere length were first averaged across fibers within each muscle/segment, and then within region (for TA).

### Ankle angle correction

Although our procedures were aimed at fixing the limbs at two selected constant ankle angles, a systematic increase (slopes: 8.7° change in ankle angle/100° change in knee angle and 15.4° change in ankle angle/100° change in knee angle for ankle angles set to 90° and 125°, respectively) in ankle angle was found with knee extension. This variation in ankle angle affects SO and TA length properties at the macroscopic (muscle belly and fiber length) and microscopic (sarcomere length) levels and should be distinguished from myofascial effects. To correct the measured muscle belly, fiber, and sarcomere lengths toward the value corresponding to the intended ankle angle of 90° or 125°, additional hindlimbs were fixed at 90° knee angle and ankle angles set to either 55° (*n* = 3) or 160° (*n* = 3). Combined with the above described hindlimbs fixed at ankle angles of 90° and 125°, this resulted in 12 muscles (for both SO and TA) fixed with the knee at 90°, and the ankle set to 55°, 90°, 125°, and 160° (three muscles for each of the ankle angles). The additional hindlimbs allowed us to fit the data over a wider range of ankle angles and, hence, to obtain a more accurate description of the ankle angle–length relationships. The muscle belly, fiber, and sarcomere lengths from these 12 muscles were fitted using a linear regression (*r*^2^ for SO > 0.9; *r*^2^ for TA > 0.7). The adjustments of muscle belly, fiber, and sarcomere lengths were made by calculating the difference in ankle angle between the target angle (either 90° or 125°) and the actual angle, based on the X-ray images. Using this difference and the slope of the fit, we could estimate the values for ankle angles of exactly 90° and 125°.

### Statistics

To assess effects of knee angle on normalized muscle belly length and normalized fiber length of SO and TA, ANCOVAs (SPSS Statistics 21, IBM Corporation, Armonk, NY) were used with ankle angle as a fixed factor and knee angle as a covariate. For SO, effects of knee angle on sarcomere length were assessed by a mixed ANCOVA design with ankle angle as a fixed factor, knee angle as a covariate, and segment (proximal, intermediate, distal) as a within subject factor. Main effects of all independent variables were assessed as well as their interactions with knee angle. For TA, effects of knee angle on sarcomere length were assessed by a mixed ANCOVA design with ankle angle as a fixed factor, knee angle as a covariate, and region (medial, lateral) and segment (proximal, intermediate, distal) as within-subject factors. Main effects of all independent variables were assessed as well as their interactions with knee angle.

## Results

### Effects of knee angle on SO muscle, fiber, and sarcomere lengths

Knee angle did not affect normalized SO muscle belly length (mean across knee angles for ankle at 90°: 24.4 ± 0.7 mm; %L_M_T_: 0.64 ± 0.02; mean across knee angle for ankle at 125°: 21.0 ± 1.1 mm; %L_M_T_: 0.55 ± 0.02), and no interaction effect with ankle angle was found (Table1). Similar results were found for SO fiber length: no main effect of knee angle (ankle at 90°: 14.9 ± 0.8 mm; %L_F_T_: 0.39 ± 0.02; ankle at 125°: 12.2 ± 0.8 mm; %L_F_T_: 0.32 ± 0.02) on normalized SO fiber length was found, nor an interaction effect between knee angle and ankle angle.

**Table 1:** Statistical results of the ANCOVAs (for normalized muscle belly length and fiber length) and repeated measures ANCOVAs (for sarcomere length) for soleus (SO) and tibialis anterior (TA)

	Muscle belly length (%L_M_T_)	Fiber length (%L_F_T_)	Sarcomere length (*μ*m)
SO			
Knee angle	*F*_1,20_ = 1.664, *P* = 0.212	*F*_1,20_ = 0.320, *P* = 0.578	*F*_1,20_ = 0.359, *P* = 0.556
Ankle angle	*F*_1,20_ = 22.443, ***P***** < 0.001**	*F*_1,20_ = 10.296, ***P*** = **0.004**	*F*_1,20_ = 32.380, ***P***** < 0.001**
Segment	–	–	*F*_2,40_ = 29.785, ***P***** < 0.001**
Knee × Ankle	*F*_1,20_ = 1.131, *P* = 0.300	*F*_1,20_ = 0.664, *P* = 0.425	*F*_1,20_ = 0.872, *P* = 0.362
Knee × Segment	–	–	*F*_2,40_ = 0.370, *P* = 0.693
Knee × Segment × Ankle	–	–	*F*_2,40_ = 0.243, *P* = 0.786
TA			
Knee angle	*F*_1,20_ = 0.000, *P* = 0.997	*F*_1,20_ = 0.077, *P* = 0.784	*F*_1,20_ = 0.078, *P* = 0.783
Ankle angle	*F*_1,20_ =18.662, ***P***** < 0.001**	*F*_1,20_ = 0.895, *P* = 0.355	*F*_1,20_ = 6.274, ***P*** = **0.021**
Region	–	*F*_1,20_ = 7.451, ***P*** = **0.013**	*F*_1,20_ = 4.252, *P* = 0.052
Segment	–	–	*F*_2,40_ = 4.410, ***P*** = **0.019**
Knee × Ankle	*F*_1,20_ = 3.582, *P* = 0.073	*F*_1,20_ = 1.525, *P* = 0.231	*F*_1,20_ = 0.205, *P* = 0.655
Knee × Region	–	*F*_1,20_ = 0.119, *P* = 0.733	*F*_1,20_ = 0.270, *P* = 0.609
Knee × Segment	–	–	*F*_2,40_ = 10.298, ***P***** < 0.001**
Knee × Region × Ankle	–	*F*_1,20_ = 2.927, *P* = 0.103	*F*_1,20_ = 0.113, *P* = 0.740
Knee × Region × Segment	–	–	*F*_2,40_ = 1.560, *P* = 0.223
Knee × Region × Ankle × Segment	–	–	*F*_2,40_ = 0.394, *P* = 0.677

*P* values refer to the significance of the statistical test for each effect, significant values are printed in bold. %L_M_T_ and %L_F_T_: muscle belly length and fiber length normalized to tibia length.

**Figure 3:**
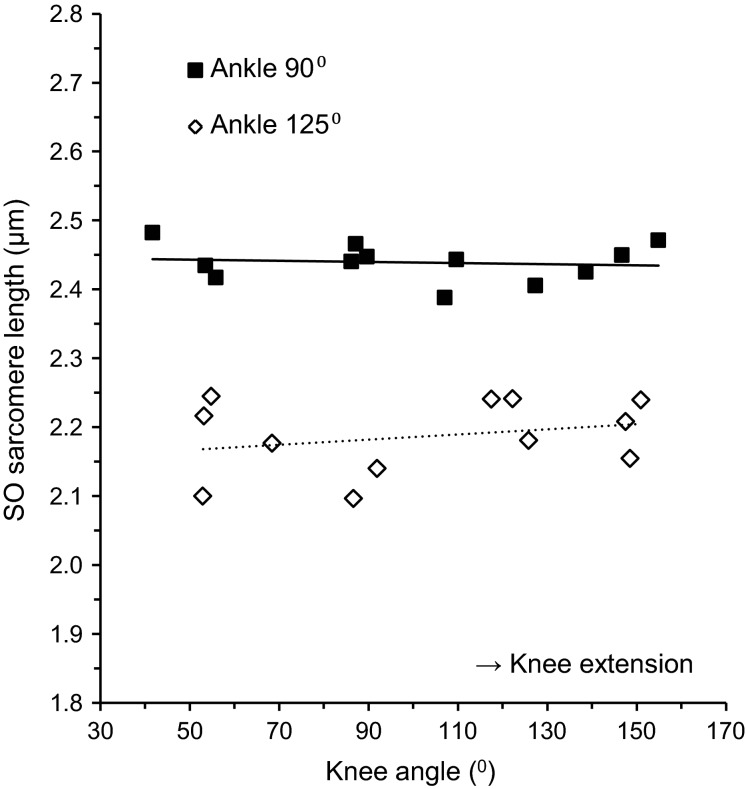
Relationship between knee angle and SO fiber mean sarcomere length with ankle angle set to 90° (■, *n* = 12) and 125° (◊, *n* = 12). Slopes of the linear regression lines were −0.01 *μ*m/100° and 0.04 *μ*m/100°, respectively. Note that each data point represents the SO fiber mean sarcomere length of one muscle.

No main effect of knee angle on SO fiber mean sarcomere length was found (Fig.3, Table1), both at 90° ankle angle (2.44 ± 0.03 *μ*m) and 125° ankle angle (2.19 ± 0.05 *μ*m). Nor did we find an interaction effect of knee angle and other factors. These results indicate that the length of SO at the macroscopic as well as the microscopic level was independent of changes in knee angle involving MTU length changes of synergistic muscles.

### Effects of knee angle on TA muscle, fiber, and sarcomere lengths

Normalized TA muscle belly length was not affected by knee angle (mean across knee angles for ankle at 90°: 26.6 ± 0.9 mm; %L_M_T_: 0.70 ± 0.01; mean across knee angle for ankle at 125°: 28.2 ± 1.5 mm; %L_M_T_: 0.75 ± 0.02), and no interaction effect with ankle angle was found (Table1). Knee angle did not affect normalized TA fiber length, and no interaction effect between knee angle and other factors was found. The fiber length in the lateral TA region (mean across knee angle for ankle at 90°: 16.4 ± 1.1 mm, %L_F_T_: 0.43 ± 0.02; mean across knee angle for ankle at 125°: 17.3 ± 1.4 mm, %L_F_T_: 0.45 ± 0.03) was higher than the fiber length in the medial TA region (mean across knee angle for ankle at 90°: 14.0 ± 0.5 mm, %L_F_T_: 0.37 ± 0.01; mean across knee angle for ankle at 125°: 15.7 ± 0.9 mm, %L_F_T_: 0.41 ± 0.02).

**Figure 4:**
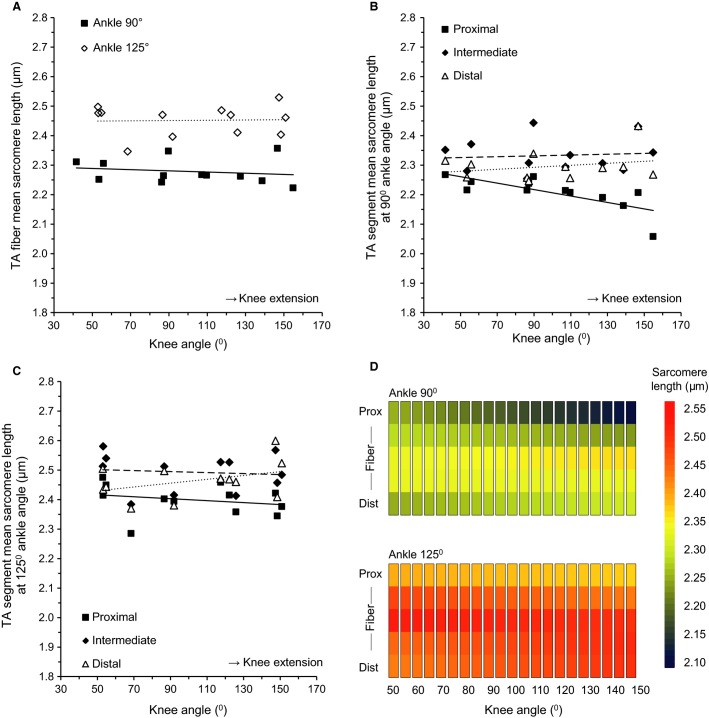
Effects of knee angle on sarcomere lengths of the tibialis anterior (TA). (A) TA fiber mean sarcomere lengths given for the pooled medial and lateral region with ankle angle set to 90° (■, *n* = 12) and 125° (◊, *n* = 12). Slopes of the linear regression lines were −0.02 *μ*m/100° and 0.00 *μ*m/100°, respectively. (B) Length of sarcomeres located in proximal (■), intermediate (♦), and distal (∆) fiber segments with ankle angle set to 90°. Slopes of the linear regression lines were −0.11 *μ*m/100°, 0.01 *μ*m/100°, and 0.03 *μ*m/100°, respectively. (C) Length of sarcomeres located in proximal (■), intermediate (♦), and distal (∆) fiber segments with ankle angle set to 125°. Slopes of the linear regression lines were −0.03 *μ*m/100°, 0.02 *μ*m/100°, and 0.06 *μ*m/100°, respectively. (D) Effects of knee angle on sarcomere length distribution in TA at ankle angles of 90° and 125°.

Although no main effect was found of knee angle on TA fiber mean sarcomere length (Table1, Fig.4A), a significant interaction effect between knee angle and segment was found (Fig.4B, C). This interaction effect was neither affected by region nor by ankle angle. The four-way interaction between knee angle, region, segment, and ankle angle was also not significant. For visualization purposes, fibers were divided into five segments (from proximal to distal) and for each segment a linear regression analysis was performed to assess the effects of knee angle (Fig.4D). The distribution as shown in Figure4B and C is easily recognized. Furthermore, it is shown that the sarcomere lengths in the middle segments are higher than at the ends, and that the sarcomere lengths in the middle segments were minimally affected by changes in knee angle. Thus, although changes in knee angle did not affect the mean length of TA at the macroscopic and microscopic levels, it did affect the distribution of sarcomere lengths at the microscopic level.

## Discussion

To the best of our knowledge, this is the first study to investigate effects of intermuscular interactions on sarcomere lengths of passive rat SO and TA muscles. The present study shows that the relative position of passive synergistic muscles, as imposed by changes in knee angle, did not affect the mean sarcomere length of SO and TA muscle fibers. However, it did result in local length changes of in-series sarcomeres, but in TA muscle fibers only.

### Effects of intermuscular interaction between passive muscles

Several studies have investigated effects of knee extension (i.e., proximal lengthening of gastrocnemius) on the length of passive SO fascicles in humans using ultrasound imaging. Results were inconsistent between studies, with decreases in SO fascicle length (Bojsen-Møller et al. 2010; Tian et al. 2012), decreased and increased (variable between subjects) fascicle lengths (Diong and Herbert 2015), and no change in fascicle length (Kawakami et al. 1998) reported. In the present study, no effects of knee extension on fiber length and fiber mean sarcomere length of SO and TA muscles were found. These somewhat contradictory results may partially be caused by unintended changes in ankle angle in some studies. Even though the ankle was tightly fixed in a brace, knee extension resulted in some ankle plantar flexion in one study (Diong and Herbert 2015). This was also found in the present study. Because changes in ankle angle result in changes in MTU length of the monoarticular muscle, a correction was performed in both the latter and present study. In the studies of Bojsen-Møller et al. (2010) and Tian et al. (2012), however, changes in ankle angle were not assessed.

Although we found a main effect of segment on SO and TA sarcomere lengths, additional measurement on SO have shown that this may be an artifact of the fixation procedure. Importantly, we found no effects of knee angle on the in-series distribution of sarcomere length for each of the segments within SO muscle fibers. This appears to be in contrast with a recent study in humans (Huijing et al. 2011) that showed with magnetic resonance imaging that knee extension caused locally both negative (shortening) and positive (lengthening) strains in SO. Although this suggests myofascial force transmission between SO and its synergists, it is important to note that the local strains calculated were not in the direction of SO muscle fibers. Considering that only strains in the muscle fiber direction affect sarcomere length, the physiologically relevant strain was likely to be smaller than reported in that study. In theory, such local strains can also be caused by changes in the pennation angle of muscle fibers without changes in sarcomere length, which could explain the discrepancy between the studies. Another reason of the discrepancy could be the fact that the architecture of the soleus muscle in humans (Hodgson et al. 2006) is quite different from that in rats (Close 1964; Eng et al. 2008; Johnson et al. 2011).

In the present study, nonuniform length changes of in-series sarcomeres were found within TA muscle fibers. The highest change in sarcomere length was ∼0.1 *μ*m for a 100° change in knee angle. If compared to fiber mean sarcomere length, this seems to be a small change (∼4%). However, it is a substantial change when compared to the changes in sarcomere length as a function of ankle joint angle (0.4 *μ*m per 100°, used to correct for deviations in ankle angle; data not shown). Hence, the changes found were ∼25% of those during ankle movement, which considering that a change in knee angle does not affect the muscle–tendon unit length of TA is substantial.

The nonuniform length changes of in-series sarcomeres within TA muscle fibers, but not within SO muscle fibers, could potentially be related to the number of in-series sarcomeres. If the number of in-series sarcomeres is lower, effects of intermuscular interactions for each sarcomere are more pronounced. On the basis of the data of the present study, we calculated the number of in-series sarcomeres and found a mean (±SD) of 5819 ± 493 sarcomeres for SO, 6101 ± 538 for the medial TA region, and 7316 ± 487 for the lateral TA region. For this reason, the number of sarcomeres in series cannot explain the different results of SO and TA. Therefore, the most likely explanation is that the epimuscular myofascial forces exerted onto TA (by, e.g., the neurovascular tract (Maas et al. 2001)) were higher than those exerted onto SO.

### Comparison with FE model of skeletal muscle

Experimental data in rats have shown that a proximal increase in the MTU length of EDL resulted in an increase in passive force measured at the tied distal tendons of TA and extensor hallucis longus (Huijing and Baan 2001, 2003, 2008). FE models suggest that these mechanical effects can be explained by local length changes of sarcomeres within the restrained muscle (Yucesoy 2010). Lengthening a neighboring muscle proximally would induce a net proximally directed load on the length-restrained muscle, which results in lower lengths of sarcomeres proximally within muscle fibers than of those located distally (Yucesoy et al. 2003). A similar length distribution of sarcomeres was found in the present study, but as a result of proximal shortening instead of lengthening of the EDL muscle (by knee extension). This suggests that the net myofascial load cannot be explained by changes in the position of EDL relative to TA. It has been found that knee extension results in proximal displacement (Ellis 2012) and lengthening strains (Restaino et al. 2014) of the sciatic nerve in humans and rats, respectively. As the common peroneal nerve, which innervates TA, branches off the sciatic nerve posterior to the knee axis, also proximal displacement of this nerve with knee extension can be expected. This may result in a proximally directed load by the neurovascular tract on TA, which would be in agreement with changes in sarcomere length distribution found in the present study.

### Implications for physiological movements

During locomotion, SO and TA muscles are active to exert force for body support and hindlimb movements. For active muscle conditions, previous studies have shown that knee angle has only very limited effects on the ankle moments exerted by SO (Tijs et al. 2015a) and TA (Tijs et al. 2015b), suggesting a limited net effect at the joint of epimuscular myofascial connections. Epimuscular myofascial force transmission per se could not be excluded in these studies. Therefore, it cannot be inferred whether changes in sarcomere distribution, found in the present study, affects the force producing capacity of the individual muscles. Furthermore, during locomotion there are also phases of the step cycle in which these muscles are passive. While SO is mainly passive during the swing phase, TA is passive during the stance phase (Sabatier et al. 2011). The results of the present study are, therefore, relevant for those phases of movements in which these muscles are passive. The local length changes of sarcomeres within passive TA muscle fibers found in the present study may be detected by sensory receptors within TA muscle belly such as muscle spindles. Recently it was indeed found that length changes of a synergistic muscle affected the firing pattern of muscle spindles within a length-restrained muscle (Smilde et al. 2014). As a result, these spindles may contain information about the angle of the knee joint while TA in fact does not span that joint. However, more research is necessary on the effects of epimuscular myofascial force transmission on muscle sensory information.

## Conclusion

Our study showed that changes in knee angle result in nonuniform length changes of in-series sarcomeres within muscle fibers of TA, but not of SO. Therefore, we conclude that, while epimuscular myofascial connections with neighboring structures are not functionally relevant in causing local sarcomere length changes within passive SO, they are effective within passive TA muscle.
